# Modes of the antibiotic activity of amphotericin B against *Candida albicans*

**DOI:** 10.1038/s41598-019-53517-3

**Published:** 2019-11-19

**Authors:** Ewa Grela, Agnieszka Zdybicka-Barabas, Bozena Pawlikowska-Pawlega, Malgorzata Cytrynska, Monika Wlodarczyk, Wojciech Grudzinski, Rafal Luchowski, Wieslaw I. Gruszecki

**Affiliations:** 1 Department of Biophysics, Institute of Physics, Maria Curie-Sklodowska University, Lublin, Poland; 2 Department of Plant Physiology and Biophysics, Institute of Biological Sciences, Faculty of Biology and Biotechnology, Maria Curie-Sklodowska University, Lublin, Poland; 3 Department of Immunobiology, Institute of Biological Sciences, Faculty of Biology and Biotechnology, Maria Curie-Sklodowska University, Lublin, Poland; 4 Department of Functional Anatomy and Cytobiology, Institute of Biological Sciences, Faculty of Biology and Biotechnology, Maria Curie-Sklodowska University, Lublin, Poland

**Keywords:** Cellular imaging, Pharmaceutics

## Abstract

Amphotericin B is an antibiotic used as the “gold standard” in the treatment of life-threatening fungal infections. Several molecular mechanisms have been proposed to explain exceptionally high effectiveness of amphotericin B in combating fungi. In the present work, we apply fluorescence lifetime imaging microscopy to track, step by step, modes of the toxic activity of amphotericin B towards a clinical strain of *Candida albicans*. The images recorded reveal that the antibiotic binds to cells in the form of the small aggregates characterized by a relatively short fluorescence lifetime (0.2 ns). Amphotericin B binds preferentially to the cell walls of mature cells but also to the plasma membranes of the daughter cells at the budding stage. The images recorded with the application of a scanning electron microscopy show that the antibiotic interferes with the formation of functional cell walls of such young cells. The results of imaging reveal the formation of the amphotericin B-rich extramembranous structures and also binding of the drug molecules into the cell membranes and penetration into the cells. These two modes of action of amphotericin B are observed in the time scale of minutes.

## Introduction

The dramatic increase in systemic fungal infections, that is observed over the last decades, is a challenge for research centres worldwide, active in elaboration of effective and safe antimycotic pharmaceuticals^1–3^. Amphotericin B (AmB) has been used as the “gold standard” to treat life-threatening mycoses, owing to the high effectiveness of this antibiotic and despite the severe toxic side effects^4^ (see Supplementary Information Fig. S1 for a chemical structure). According to our current understanding, biomembranes are a primary target of AmB in both the pharmacologically desirable and the toxic side effects of the antibiotic. Formation of transmembrane pores able to act as ion channels interfering with the cell electrostasis is considered as one of the main mechanisms underlying the biological activity of AmB^5–8^. The fact that such intramembranous structures are more readily formed in the membranes containing ergosterol, a sterol of fungi, than in the case of cholesterol that is present in mammalian cells, is a central paradigm of AmB selectivity. A substantially different kind of the biological activity of AmB also was demonstrated, relying in destabilization of biomembranes realized via extraction of sterols which were immobilized within extramembranous, two-component sterol-AmB sponge-like structures^9^. Interestingly, formation of such cholesterol-AmB structures, in the case of mammalian cells exposed to AmB, was demonstrated to have a potent protective effect against toxicity of the drug^10^. Very recently, another self-protective strategy of human cells against AmB toxicity has been reported, associated with the formation of AmB-rich exosomes eliminating molecules of the drug from the plasma membranes^11^. In the light of all the recent findings, listed above, a picture regarding the biological effects of AmB on biomembranes looks more complex than generally expected. In the present work, we apply a technique of fluorescence lifetime imaging microscopy (FLIM), which gives insight into the molecular organization and localization of AmB, to track and analyse different modes of the activity of the drug with respect to the popular, clinical strain of fungi: *Candida albicans* (referred to as *Candida*).

## Results

**Figure 1:**
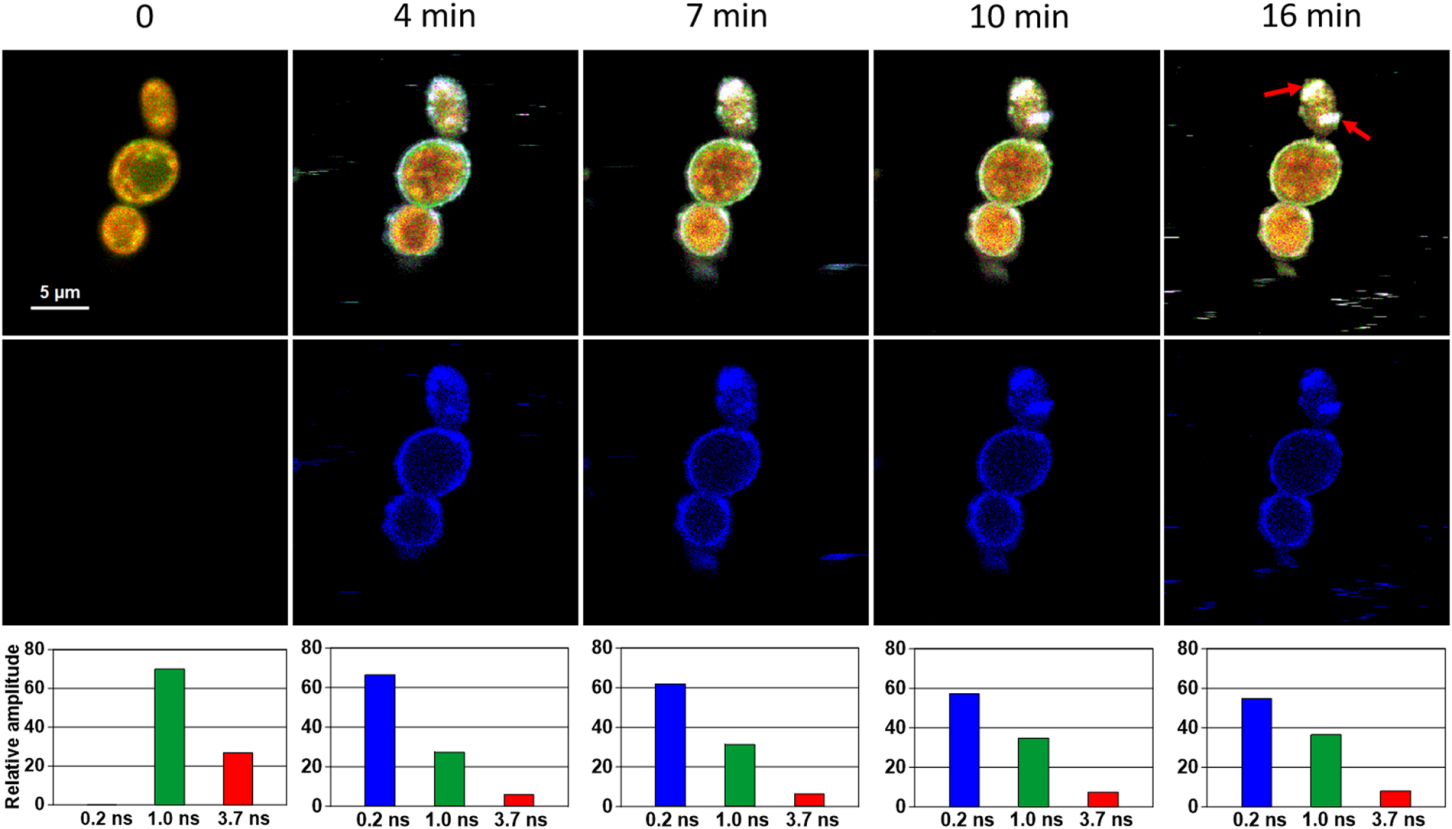
Fluorescence lifetime images of cells. Cells of *Candida albicans* were imaged with FLIM technique before (time 0) and after the injection of amphotericin B. Images were recorded after the time periods indicated at the top of each image. The colour codes of the images: blue - the fluorescence lifetime component 0.2 ns, green - the fluorescence lifetime component 1.0 ns, red – the fluorescence lifetime component 3.7 ns. The middle panel presents the same images as the top panel but with displayed exclusively the short-lifetime component representing the presence of AmB in the objects imaged. The lowest panel presents relative amplitudes of the fluorescence lifetime components determined for individual images. A final concentration of AmB in the sample 100 µM, concentration of DMSO 0.3%. An effective AmB concentration for the experiments has been selected on the basis of the results of cell viability tests (shown in the Supplementary Fig. S2). The exemplary original fluorescence lifetime kinetic traces are presented in the Supplementary Fig. S3. Selected bulk structures that appeared after exposure of the cells to AmB are marked with red arrows.

**Figure 2:**
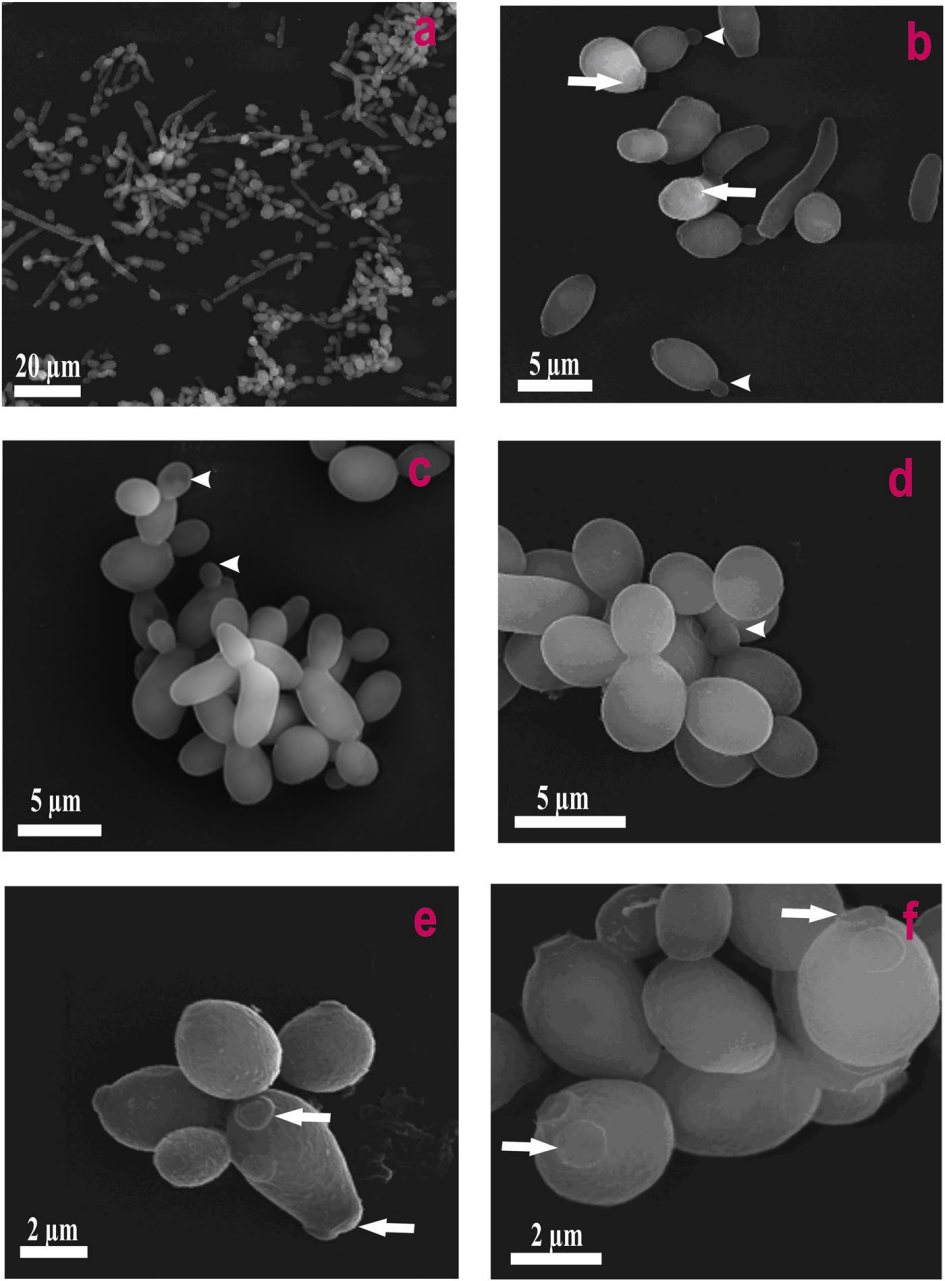
Scanning Electron Microscopy images of control *Candida albicans* cells. (**a**) The *C. albicans* from the culture. Note the prevalent presence of spherical, ovoid-shaped and rare of long, extended hyphal filaments. (**b**) The cells show singular buds (arrowheads) and scars located at the tips. Some cells develop multiple scars located at the pole or randomly positioned (arrows). (**c**) Budding forms of the elongated cells. (**d**) Ovoid cells with buds (arrowhead) and bud scars located at the tips. (**e**,**f**) Enlargement view of the cells with spherical shape and bud scars in multiple or singular forms (arrows) located mainly at the poles.

**Figure 3:**
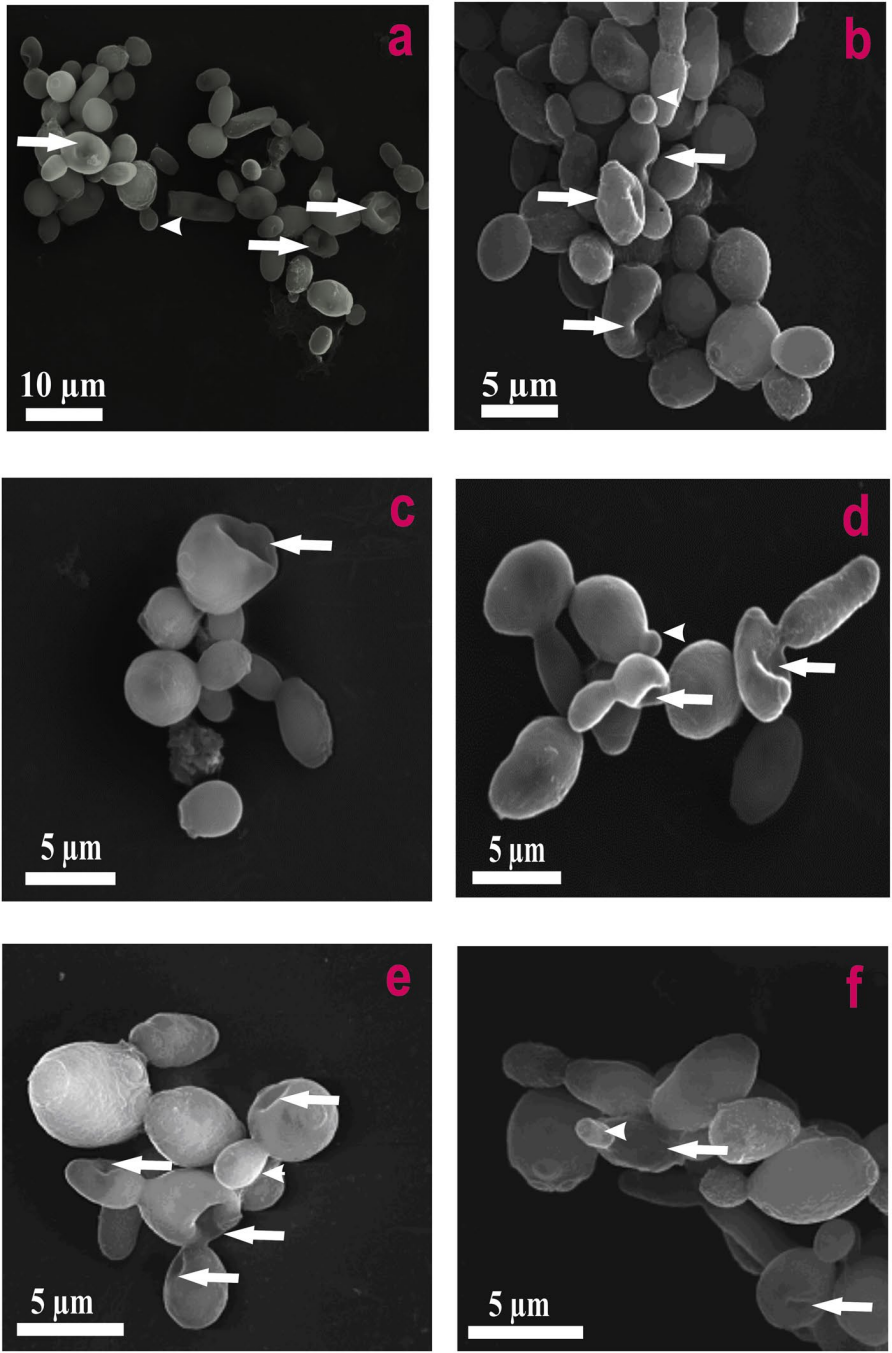
Characteristics of the surface phenotype of *C. albicans* after treatment with AmB. (**a,b**) Micrographs showing altered cells after incubation with AmB. Cells with indentations (arrows) can be seen. Among ovoid cells elongated yeast cells are discernible. Arrowhead indicates small bud. (**c,d**) Collapsed mother cells with indentations are shown (arrows). Note the presence of emerging buds at some cells (arrowheads). (**e**) Affected spherical mother cells, as well as newly formed budding, are seen (arrows). Note the presence of the cell with no changes to new bud (arrowhead). (**f**) The cells showing some deformed phenotype (arrows). Arrowhead indicates emerging bud with irregular shape.

**Figure 4:**
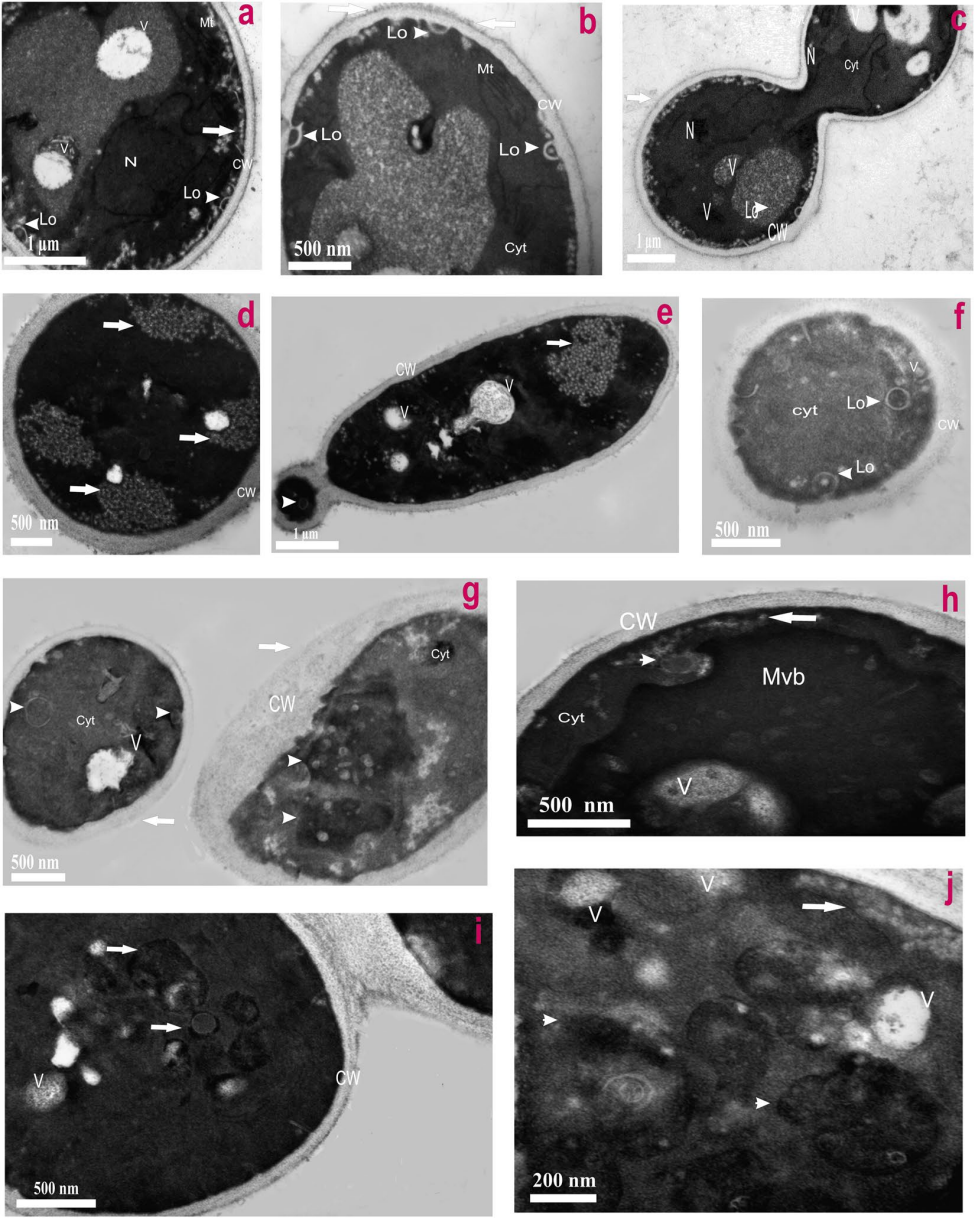
The influence of amphotericin B on *C. albicans* cell ultrastructure. Control cells (**a**–**c**) and amphotericin B treated cells (**d**–**j**). (**a**) Fragment of control cell showing well preserved structure with distinct nucleus (N), mitochondrion (Mt), lomasomes (arrowheads- Lo), big vacuoles (V) and cell wall (CW); (**b**) Prominent brush-like material adherent to cell wall (arrows); (**c**) Dividing cell with visible big vacuoles (V), lomasomes (Lo) and nucleus (N); (**d**,**e**) AmB treated cell in which aggregations of tiny vacuoles are seen (arrows). Note the presence of separated lomasome inside small emerging cell (arrowhead); (**f**) separated lomasomes in the cell with thick cell wall (arrowheads); (**g**) the left-hand side cell with disconnected lomasomes (arrowhead) and in right-hand side cell distinct multivesicular bodies - like structures (Mvb) are seen (arrowhead); (**h**) the cell with visible Mvb in cytoplasm; (**i**) in, emerging from mother cell, daughter cell vesicular structures are visible (arrows) in dense cytoplasm; (**j**) enlarged portion of cytoplasm from young cell with small vacuoles (arrows) close to cell wall, big vacuole (V) and vesicular - like bodies (arrowheads).

**Figure 5:**
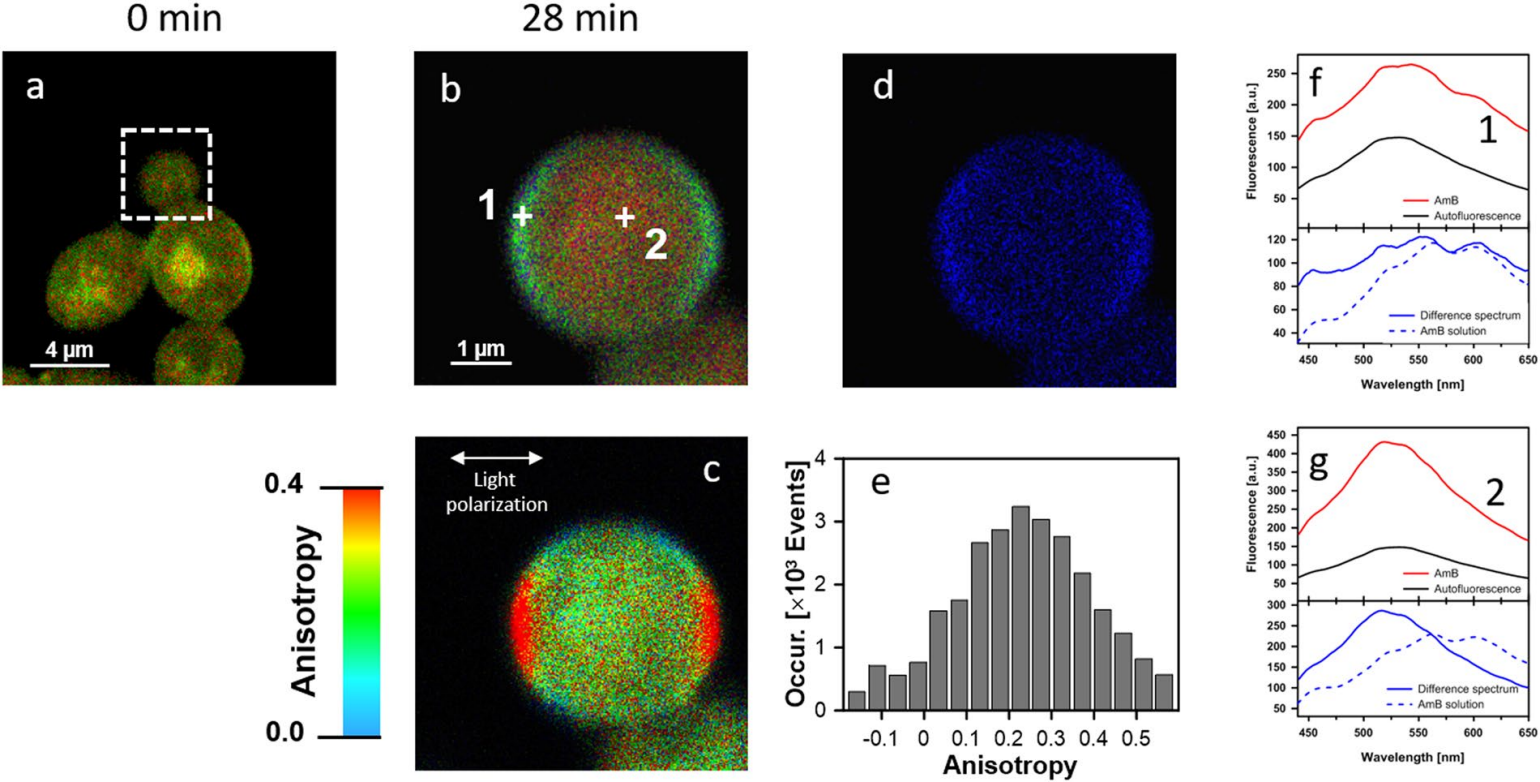
Fluorescence lifetime images of cells. Cells of *Candida albicans* were imaged with FLIM technique before (time 0) and 28 min after the injection of amphotericin B (indicated). A final concentration of AmB in the sample 100 µM. The concentration of DMSO 0.3%. Panels b, c and d present magnification of the young cell, at the budding state, marked in panel a with a white square. (**b**) FLIM image with indicated points in which the fluorescence emission spectra (white cross, spectrum 1 and spectrum 2) presented in panels f and g were recorded. (**c**) Fluorescence anisotropy image. (**d**) Image created based exclusively by the short fluorescence lifetime component representing amphotericin B. (**e**) Distribution of fluorescence lifetime anisotropy from image presented in panel c. (**f**) Fluorescence emission spectra recorded from the cell exposed to AmB (red) and autofluorescence spectrum recorded in time 0 (black). The lower panel presents the difference spectrum calculated by subtraction of the spectra presented in the upper panel (solid blue line) superimposed on the spectrum recorded from the water phase outside the cell after injection of AmB. (**g**) legend the same as in the case of panel f except that the spectra were recorded in the position 2.

**Figure 6:**
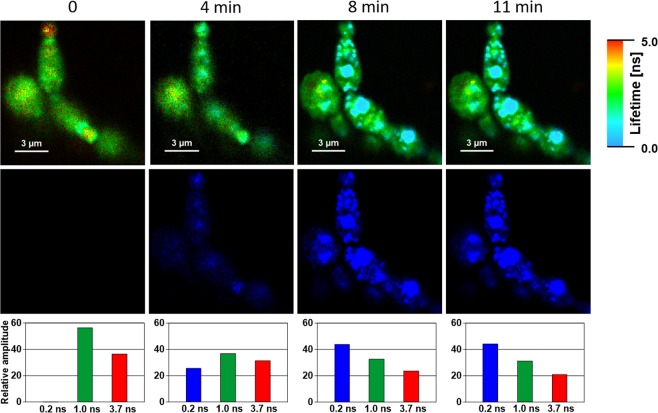
Fluorescence lifetime images of cells. Cells of *Candida albicans* were imaged with FLIM technique before (time 0) and after the injection of amphotericin B. Images were recorded after the time periods indicated at the top of each image. The colour codes of the images: blue - the fluorescence lifetime component 0.2 ns, green - the fluorescence lifetime component 1.0 ns, red – the fluorescence lifetime component 3.7 ns. The middle panel presents the same images as the top panel but with displayed exclusively the short-lifetime component representing the presence of AmB in the objects imaged. The lowest panel presents relative amplitudes of the fluorescence lifetime components determined for individual images. A final concentration of AmB in the sample 300 µM, concentration of DMSO 1.3%.

Figure 1 presents the FLIM images of *Candida* cells before and after the exposition to AmB. Images recorded after different periods of time following the antibiotic injection into the assay, allow an analysis of binding of molecules of AmB to cells and evolution of the system. Natural autofluorescence of *Candida* cells is represented by the two lifetime components represented in the images presented in Fig. 1 by green colour code (1.0 ns) and by red colour code (3.7 ns). The relative amplitudes of those lifetime components are presented in the lowest panel of Fig. 1. Similarly to the experiments carried out with model lipid membranes^12,13^ and cells from the human cell cultures^11^ AmB binds to the cells in the form of small aggregated structures characterized by the relatively short fluorescence lifetime component of 0.2 ns, represented by a blue colour code. Binding of AmB to cells can be followed in Fig. 1 in the middle panel images created based exclusively on the 0.2 ns fluorescence lifetime component. As can be seen, AmB binds preferably to the cell walls, without effectively crossing this barrier covering the cell membrane. Immobilization of AmB at the cell wall can be naturally rationalized taking into account numerous polar groups of the macrolide ring of the antibiotic, which can form hydrogen bonds with polar groups of chitin, β-glucan or mannoproteins being the major constituent of the fungal cell wall^14,15^. As can be seen from Fig. 1, most of the cell structures imaged by AmB anchored in the cell wall do not undergo significant structural modification within the time period of the experiment. Interestingly, the exception can be observed in the case of the relatively small cell at the top, identified as a growing bud daughter cell. As can be seen, the concentration of AmB in this particular cell increases. Moreover, one can observe the formation of the bulk AmB-rich structures at the cell surface and eventually, morphological changes of the entire cell. Such an observation suggests that AmB can more readily pass the cell wall barrier of young cells at the budding stage. Such a mechanism can be understood on the basis of affected integrity of the cell wall in a course of cell budding and on the basis of decreased rigidity of the lipid bilayer throughout this process^16^. Formation of extracellular bulk structures in the *Candida* cultures exposed to AmB, combined with pronounced morphological alterations of cells can also clearly be visible by means of scanning electron microscopy (SEM, compare Figs 2 and 3). *Candida albicans* is a polymorphic fungus that morphologically has several different forms. In our investigation, SEM technique was applied to analyze morphology of *C. albicans* cells under the influence of AmB. Control cells had mostly spherical, ovoid-shaped budding cells with smooth walls (Fig. 2a,d–f). Among them, occasionally, extended tube-like blastoconidia were also noted (Fig. 2b,c). Most of spherical blastoconidia had polarly located buddings. The rings of scars (remaining after offspring cells had dropped out from the mother cell) were located at the tips (poles) of the cells. SEM observations revealed also the presence of the cells with multiple scars located at the cell pole (Fig. 2e,f). Some cells had singular bud scars (Fig. 2b,e). AmB treated cells exhibited several morphological changes (see Fig. 3). Incubation of the cells with AmB for 30 min caused less spherical cells appearance. The cells were elongated to some extent and tube-like cells formation was noted. SEM analysis also revealed that oval blastoconidial mother cells that remained, had indentations (Fig. 3a–e). Collapsed cells were also found. Sometimes buddings were deformed (Fig. 3e,f). The SEM observations made in the current study, clearly confirm fungicidal action exerted by AmB. As a polyene antibiotic it can cause permeability changes that in turn result in osmotic imbalance. Hence the observed collapsed cells and indentations of the walls could be explained by this phenomenon. Cells of *C. albicans* exposed to AmB were additionally analyzed with application of Transmission Electron Microscopy (TEM, see Fig. 4). The cells from control cultures have well discernible cell wall. The characteristic feature of the cell wall is an outer brush-like layer and amorphous one consisting of short oligomannan fibrils that modify cell wall surface proteins^17,18^. The cell membrane is attached to the innermost layer of the cell wall. In close proximity of the cell membrane lomasome-like structures and small vacuoles are visible. In the cytoplasm of the control cells typical organelles for the fungal cells like round-lobate nuclei, mitochondria, and big vacuoles are seen (Fig. 4a–c). When *C. albicans* cells were exposed to AmB for 30 min some changes in the cell ultrastructure were observed. The presence of groups consisting of many small vacuoles located in peripheral part of the cell or inside the mature cells was noted (Fig. 4d,e). Additionally, in newly formed cell separated lomasomes in the form of vesicular bodies were also found near the cell membrane or deeper in cytoplasm (Fig. 4e–h). In some cells amorphous layer of the cell wall was not clearly discernible (Fig. 4g). Importantly, in the cell emerging from adult cells also multivesicular bodies-like structures were noted (Fig. 4g–j). The fact that morphological changes are observed in the cells at the budding stage, exposed to AmB, implies that the antibiotic interferes with formation of fully functional cell walls. The extracellular AmB-rich bulk structures can represent the so-called AmB-sterol “sponges” demonstrated to be responsible for ergosterol sequestration from fungal cells^9^. Alternatively, the structures observed in the present study can represent exosomes, AmB-rich lipid vesicles extruded out of the membranes as an element of self-defence detoxification strategy, reported in the case of the human cells^11^. In order to sort out what kind of structures are formed in the case of *Candida*, we analysed FLIM images of the cells exposed to AmB (see Supplementary Fig. S4), along with the analysis of fluorescence anisotropy (see Supplementary Fig. S5). As can be seen, the fluorescence anisotropy image of a single extracellular bulk structure, rich in AmB (Fig. S5) represents the homogenous distribution of the antibiotic molecules (uniform green colour-coded structures), unlike the red colour-coded structures typical for AmB bound to the lipid membranes and characterized by relatively high fluorescence anisotropy^11,13^. This indicates that the bulk forms observed in the case of *Candida* cells exposed to AmB are most probably the AmB-sterol, sponge-like structures but not exosomes observed in the case of human cells. In contrast, a detailed fluorescence anisotropy analysis of the young cell at the budding stage (see Fig. 5) reveals vertical orientation of AmB molecules with respect to the cell membrane, similar to that one observed in model lipid vesicles^13^ and exosomes that emerged from the human cells^11^. This is manifested by the relatively high fluorescence intensity and anisotropy in the cell membrane regions located in the left hand and right-hand sides of the cell imaged with the laser beam polarized as indicated in the graph (Fig. 5c). Such a result indicates binding of AmB molecules to cell membranes of *Candida*. Comparison of the fluorescence emission spectra recorded from selected compartments of a single cell (Fig. 5b,f,g) reveals different molecular organization of AmB in the cell membranes and present in the cytoplasm. The fluorescence emission spectrum of molecules present in the cell membrane is similar to the spectrum of the drug in the aggregated state, present in the water medium (Fig. 5f, lower panel) but different from the spectrum recorded inside the cell (Fig. 5g, lower panel). Comparison of those spectra with the spectra of different molecular organization forms of AmB^19^ leads to the conclusion on a heterogeneous organization of molecules of the drug in the *Candida* cells. The kinetics of the penetration of AmB into the cell can be followed in the Supplementary Material animation presenting time development of the short fluorescence lifetime component. The AmB fraction inside a cell was found to be always higher in the case when the antibiotic was present at relatively high concentrations (see Fig. 6 and Supplementary Fig. S6). In such cases, we have observed the gradual appearance of structures rich in AmB inside the cells. The time evolution of the system can be described in terms of a two-phase mechanism: formation of relatively small spherical structures within the cell regions adjacent to the plasma membrane followed by a fusion of such structures with themselves, as well as with the internal membranes of the cells, including numerous vacuole membranes. A transfer of AmB from the extracellular environment to vacuoles of fungi is a mechanism reported to take place in *Candida albicans*^20^ and *Saccharomyces cerevisiae*^21^.

## Discussion

**Figure 7:**
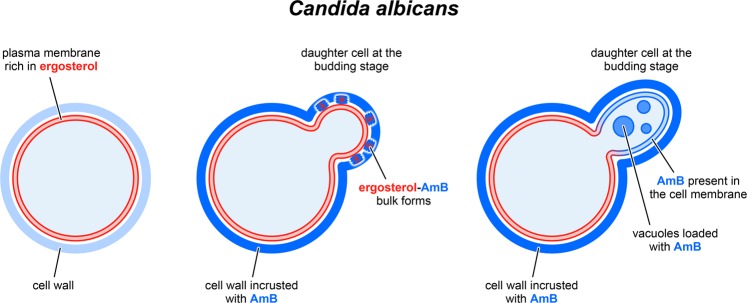
A model of activity of amphotericin B towards *Candida albicans*. Cells of *Candida albicans* (on the left) exposed to AmB (cells on the right). The modes of the biological activity of AmB towards *C. albicans* cells, presented in this simplified model, are based on the images presented in this work. It is schematically depicted that: (i) AmB binds to the cell walls of mature cells, (ii) the antibiotic prevents the formation of a functional cell wall of daughter cells at the budding stage, (iii) the antibiotic is located either in ergosterol-AmB bulk extramembranous structures or binds into the cell membrane and penetrates into the cell.

In the present work, we analysed the activity of molecules of antifungal antibiotic AmB towards *Candida albicans* with the application of the experimental approach that enables simultaneous monitoring of molecular organization and localization of the drug in the cells, based on specific spectroscopic signatures^11,13^. The results based on FLIM imaging reveal a relatively quick binding of AmB to fungal cells: on a time scale of minutes. Spectroscopic analysis of fluorescence emitted selectively by the drug molecules bound to the imaged cells shows that the biologically active AmB appears in the form of small supramolecular assemblies characterized by the lifetime component of 0.2 ns^19^ A similar molecular organization of AmB has been deduced in the case of model lipid membranes^12,13^ and the plasma membranes of the human cells^11^. Most probably, such an organization is determined by the water phase from which molecules of AmB approach examined cells. On the other hand, comparison of the fluorescence emission spectra of AmB in the water medium outside the cell, present within the cell membrane and present inside the *Candida* cell (Fig. 5) shows differences in the molecular organization of AmB, depending on the environment. As can be concluded from the image analyses, the cell wall of fungi acts as a barrier protecting the plasma membrane against interaction with AmB. On the other hand, molecules of the antibiotic can readily access the plasma membranes of cells at the budding stage, most probably owing to a cell wall loosening associated with its remodelling. The results of the experiments reported in the present work show unequivocally operation of two essentially different molecular mechanisms. Both the mechanisms potentially involved in biological activity of AmB. The first mechanism is based upon the formation of extracellular sponge-like structures and the second on the formation of intracellular AmB-rich vesicular bodies targeting vacuoles and possibly other organelles. Based on the results of TEM imaging of *Candida* cells exposed to AmB, such structures can be identified as intracellular lomasomes. Interestingly, based on our observations, we can conclude that the two modes of biological activity of AmB with respect to *Candida* cells operate on the principle of “either one or the other”. The formation of the sponge-like structures has not been observed in the cells that display intracellular accumulation of AmB (see Figs 5 and 6) and vice versa, formation of extracellular AmB-rich bulk forms has been observed in the case of the cells which do not accumulate the antibiotic in vacuoles (Fig. 1, Fig. S4). Therefore, it can be concluded that formation of extramembranous AmB-sterol structures prevents the antibiotic molecules from penetration into the cells. A very similar mechanism has been demonstrated in cells with expressed ABCA1 transporter playing a crucial role in redistribution and efflux of cholesterol in mammalian cells^10^. This indicates that trapping AmB within the extracellular bulk structures provides a mean of self-defence against toxic activity towards cells. The question is open as to how effective is such a mechanism and why in some cases AmB can penetrate the cellular membrane and realize its toxic activity? According to the literature, particularly active can be AmB transfer to vacuoles in fungi cells under a nutrient-starved condition^22^ and upon the presence of certain small molecules able to bind to biomembranes, such as allicin^20^. This indicates that a modification of structural and dynamic properties of biomembranes of fungi may affect the membrane functionality associated with the ergosterol mobility and possibly decrease the potential of molecular mechanisms associated with overall self-defence strategy against the antibiotic activity. In order to eliminate possible fluorescence originating from the *Candida* nutrient-rich growing medium, cells were transferred to a buffer directly before the experiments. Apparently, such experimental conditions were suitable for the expression and observation of different modes of the biological activity of AmB towards *Candida*. Importantly, the penetration of AmB into the cells was observed at a relatively high concentration of AmB (300 µM, Fig. 6, Fig. S6). Such a result goes along with the observation that molecules which potentially affect functionality of biomembranes, such as allicin^20^, facilitate penetration of AmB into cells. The fact that the self-protective activity of fungi against toxicity of AmB can be modulated and even inhibited opens an avenue for engineering a pharmacological formula or a treatment strategy in which AmB efficacy towards fungi would be enhanced. The modes of the biological activity of AmB with respect to *Candida albicans* cells are schematically summarized in Fig. 7. The graph presents two lines of defence of fungi cells against toxicity of AmB: (1) immobilization of AmB molecules by the cell wall and (2) binding of AmB molecules within the extracellular bulk structures. The results of the present study also allow to identify the modes of biological activity of AmB towards *Candida albicans* cells: (1) binding into the cell membrane of young cells at the budding stage, potentially affecting the membrane structural properties and physiological ion transport and (2) penetration into the cytoplasm, potentially affecting numerous intracellular organelles and physiological processes.

## Methods

### Chemicals and preparation

Antibiotic amphotericin B from *Streptomyces* sp. and dimethyl sulfoxide (DMSO) were purchased from Sigma Aldrich (USA). All other chemicals used in the preparations were of analytical grade. Water was purified by a Milli-Q system from Merck Millipore (France).

In order to additionally purify AmB, a powder of the antibiotic was suspended in a mixture of water and chloroform (1:1, v/v) and vortexed for 30 min, then collected from the interphase (this procedure has been repeated three times)^19,23^. Finally, AmB was evaporated under gaseous nitrogen.

A concentration of the antibiotic was determined based on a molar extinction coefficient of AmB in DMSO solution (121 400 M^−1^ cm^−1^) at 416 nm absorption maximum^24^. For this purpose, electronic absorption spectra were recorded with the application of a Cary 60 UV-Vis spectrophotometer from Agilent Technologies (Australia).

### Yeast strains

The yeast *Candida albicans* (wild-type; kindly gifted by Prof. A. Kędzia, Department of Oral Microbiology, Medical University of Gdansk, Poland) was cultivated in a liquid YPD medium (1% yeast extract, 2% peptone, 2% dextrose) at 37 ◦C. In the experiments, yeast in the logarithmic phase of growth were used^25,26^.

### Viability assays

The *C. albicans* survival rate after the treatment with AmB was determined using a colony-counting assay, essentially as described previously^25,26^. In brief, the log-phase intact *C. albicans* cells (200 µl; OD600 = 0.2; approx. 5.4 × 10^5^CFU) suspended in YPD medium, were incubated without (control 1) and with 0.3% DMSO (control 2) or in the presence of different concentrations of AmB (final concentration 1.8–300 μM) for 0.5 h at 37 °C. Then serial dilutions were prepared, the cells were plated onto solid YPD medium, and after 24 h incubation at 37 °C the grown colonies were counted. The number of colony-forming units (CFU) was determined. The controls defined the total (100%) survival of *C. albicans* cells in the experimental conditions. Amphotericin B dilutions were prepared in 0.3% DMSO. The results represent the mean of three independent experiments, each performed in triplicate^25,26^.

### Scanning electron microscopy (SEM) of *Candida albicans* cells

The *C. albicans* cells were incubated at 37 °C for 30 min without (control) or with AmB at the concentration of 20 µM. Next, the cells were fixed with 4% glutaraldehyde in 0.1 M phosphate buffer (pH 7.2) for 2 h at a low temperature (4 °C). Then, the samples were carefully washed with 0.1 M phosphate buffer (pH 7.2). Post-fixation was carried out for 2 h, with 1% osmium tetroxide at 4 °C. Then the cells were rinsed with 0.1 M phosphate buffer (pH 7.2). After washing, dehydration in the series of ethanol gradients: 30%, 50%, 70%, 90%, and 100% (each for 10 min), was performed. Subsequently, the specimens were chemically dried with application of 98% hexamethyldisilazane (HMDS). Finally, the specimens were coated with gold in Emitech K550X Sputter Coater. The samples were imaged with TESCAN vega 3 LMU scanning electron microscope (Czech Republic).

### Transmission electron microscopy (TEM) imaging of *Candida albicans* cells

The log-phase *C. albicans* cells in ten-times diluted YPD medium (OD_600_ = 0.2) were incubated at 37 °C for 30 min (control) or with AmB (final concentration 20 µM). Then, the cells were fixed with 4.0% glutaraldehyde in 0.1 M cacodylate buffer pH 7.2 with the addition of 0.8 M sorbitol and 5.5 mM CaCl_2_, for 2 h at 4 °C that was followed with rinsing in 0.1 M cacodylate buffer. The next step was further fixation with 1.5% potassium permanganate in water for 1.5 h at 4 °C. After rinsing several times with deionized water, the cells were contrasted in 1% uranyl acetate for 1 h and dehydrated with 30%, 50%, 70%, 90%, and 100% ethanol. Eventually, the cells were embedded in LR White Resin and polymerized at 55 °C overnight. Ultrathin section (65 nm) were obtained after cutting with diamond knife. Then the sections were collected on copper grids and stained with 1% uranyl acetate for 9 min followed by Reynolds reagent (lead nitrate and sodium citrate) for 5 min. All sections were examined with electron microscope. TEM measurements were carried out in the laboratory of Electron Microscopy of the Nencki Institute of Experimental Biology of the Polish Academy of Sciences in Warsaw with application of the transmission electron microscope JEM 1400 (JEOL Co. Japan), equipped with a Roentgen microanalyzer (EDS INCA Energy TEM, Oxford Instruments, Great Britain) and a microscopic tomography system along with the CCD MORADA G2 (EMSIS GmbH, Germany) purchased from structural funds of UE within the project CZT BIM – “Equipping the biological and medical imaging laboratory”.

### Fluorescence lifetime imaging microscopy (FLIM)

For FLIM analysis, the log-phase *C. albicans* cells (200 μl of suspension; OD600 = 0.2) in diluted YPD medium was centrifuged (6000 × *g*, 10 min, 24 °C). The pellets obtained after centrifugation were washed three times with 20 mM phosphate buffer, pH 7.4 (500 μl) and finally the yeast cells were suspended in 200 μl of 20 mM phosphate buffer, pH 7.4^26^. Next, 20 μl of the resulting suspension of *C. albicans* cells (without AmB) were applied on a polylysine-coated coverslip and were imaged with FLIM technique, and then 10 μl AmB (final concentration 100 μM, 200 μM, 300 μM) was carefully added. The analyses were carried out for 30 min.

Fluorescence lifetime imaging was carried out on MicroTime 200 (Picoquant GmbH, Germany) linked with an Olympus IX71 inverted microscope. The technical details of imaging were reported previously^11^. Briefly, the cells were scanned with the 405 nm pulsed laser (10 MHz repetition frequency and 16 ps resolution time). A silicon oil-immersed objective (NA = 1.3, 60×) was used. Measurements were carried out with the application of a confocal pinhole of 50 μm in diameter and ZT 405RDC dichroic, ZET405 StopLine Notch Filter, 430 long wavelength-pass filters from Chroma-AHF Analysentechnik. The fluorescence signal was divided into perpendicular- and parallel-polarized channels and was simultaneously measured by two twin Single Photon Avalanche Diodes. Fluorescence lifetimes and fluorescence anisotropy values were analysed and determined with the application of SymPhoTime 64 v. 2.3 software (Picoquant GmbH, Germany). Fluorescence anisotropy was calculated according to the formula:

$$r=\frac{{F}_{\Vert }-G{F}_{\perp }}{{F}_{\Vert }+2G{F}_{\perp }}$$

where F_⊥_ denotes perpendicular and F_||_ parallel intensities. Polarization directions are referred to the polarization of excitation laser beam. G was an instrumental correction factor (typically 1.01). A value of factor G was determined before each experiment, in separate measurement carried out with a long-lifetime fluorescence probe.

Fluorescence emission spectra were recorded from imaged cells by spectrograph Shamrock 163 attached to the microscopy system. The detection was based on Newton EMCCD DU970P BUF camera (Andor Technolgy) cooled to −50 °C.

All type of the microscopic analyses were repeated at least for 20 different samples and typical results are presented in the manuscript and in the Supplementary Information.

## Supplementary information

Supplementary information

## References

[1] Casadevall, A. Emerging fungal pathogens—past, present, and future. in *Public workshop, “Fungal Diseases: An Emerging Challenge to Human, Animal and Plant Health” Forum on Microbial Threats* (Institute of Medicine, Washington, D.C., 2010).

[2] Olsen, L.A., Choffnes, E.R., Relman, D.A. & Pray, L. (eds.). *Fungal diseases: an emerging threat to human, animal, and plant health*, (The National Academies Press, Washington, DC, 2011). 22259817

[3] Srivastava V, Singla RK, Dubey AK (2018). Emerging Virulence, Drug Resistance and Future Anti-fungal Drugs for Candida Pathogens. Curr Top Med Chem.

[4] Mora-Duarte J (2002). Comparison of caspofungin and amphotericin B for invasive candidiasis. N. Engl. J. Med..

[5] De Kruijff B, Demel RA (1974). Polyene antibiotic-sterol interaction in membranes of Acholeplasma laidlawii cells and lecithin liposomes; III Molecular structure of the polyene antibiotic-cholesterol complex. Biochim. Biophys. Acta.

[6] Ermishkin LN, Kasumov KM, Potzeluyev VM (1976). Single ionic channels induced in lipid bilayers by polyene antibiotics amphotericin B and nystatine. Nature.

[7] Matsuoka S, Murata M (2003). Membrane permeabilizing activity of amphotericin B is affected by chain length of phosphatidylcholine added as minor constituent. Biochim. Biophys. Acta..

[8] Neumann A, Czub J, Baginski M (2009). On the possibility of the amphotericin B-sterol complex formation in cholesterol- and ergosterol-containing lipid bilayers: A molecular dynamics study. J. Phys. Chem. B.

[9] Anderson TM (2014). Amphotericin forms an extramembranous and fungicidal sterol sponge. Nat. Chem. Biol..

[10] Wu, A. *et al*. ABCA1 transporter reduces amphotericin B cytotoxicity in mammalian cells. *Cell Mol Life Sci*, 10.1007/s00018-019-03154-w (2019). 10.1007/s00018-019-03154-w PMC6881254 31134303

[11] Grela E (2018). Imaging of human cells exposed to an antifungal antibiotic amphotericin B reveals the mechanisms associated with the drug toxicity and cell defence. Sci Rep.

[12] Grela E (2018). A mechanism of binding of an antifungal antibiotic amphotericin B to lipid membranes: An insight from combined single membrane imaging, micro-spectroscopy, and molecular dynamics. Mol. Pharm..

[13] Grudzinski W, Sagan J, Welc R, Luchowski R, Gruszecki WI (2016). Molecular organization, localization and orientation of antifungal antibiotic amphotericin B in a single lipid bilayer. Sci. Rep..

[14] Lenardon MD, Munro CA, Gow NAR (2010). Chitin synthesis and fungal pathogenesis. Current Opinion in Microbiology.

[15] Gow NAR, van de Veerdonk FL, Brown AJP, Netea MG (2012). Candida albicans morphogenesis and host defence: discriminating invasion from colonization. Nature Reviews Microbiology.

[16] Banerjee P, Pal S, Kundu N, Mondal D, Sarkar N (2018). A cell-penetrating peptide induces the self-reproduction of phospholipid vesicles: understanding the role of the bilayer rigidity. Chemical Communications.

[17] Masuoka J (2004). Surface glycans of Candida albicans and other pathogenic fungi: Physiological roles, clinical uses, and experimental challenges. Clinical Microbiology Reviews.

[18] Goswami RR, Pohare SD, Raut JS, Karuppayil SM (2017). Cell surface hydrophobicity as a virulence factor in *Candida albicans*. Biosci. Biotech. Res. Asia.

[19] Starzyk J (2014). Self-association of amphotericin B: spontaneous formation of molecular structures responsible for the toxic side effects of the antibiotic. J. Phys. Chem. B.

[20] Borjihan H, Ogita A, Fujita K, Hirasawa E, Tanaka T (2009). The vacuole-targeting fungicidal activity of amphotericin B against the pathogenic fungus Candida albicans and its enhancement by allicin. Journal of Antibiotics.

[21] Kang CK (2013). Visualization analysis of the vacuole-targeting fungicidal activity of amphotericin B against the parent strain and an ergosterol-less mutant of Saccharomyces cerevisiae. Microbiology-Sgm.

[22] Yoshioka M (2016). The fungicidal activity of amphotericin B requires autophagy-dependent targeting to the vacuole under a nutrient-starved condition in Saccharomyces cerevisiae. Microbiology-Sgm.

[23] Wasko P (2012). Toward understanding of toxic side effects of a polyene antibiotic amphotericin B: Fluorescence spectroscopy reveals widespread formation of the specific supramolecular structures of the drug. Mol. Pharmaceut..

[24] Hargreaves PL, Nguyen TS, Ryan RO (2006). Spectroscopic studies of amphotericin B solubilized in nanoscale bilayer membranes. Biochim. Biophys. Acta.

[25] Sowa-Jasilek A (2014). Studies on the role of insect hemolymph polypeptides: Galleria mellonella anionic peptide 2 and lysozyme. Peptides.

[26] Sowa-Jasilek A (2016). Galleria mellonella lysozyme induces apoptotic changes in Candida albicans cells. Microbiological Research.

