# Supplementary material for: Modes of the antibiotic activity of amphotericin B against Candida albicans
Source: Sci Rep. 2019 Nov 19;9:17029. doi: 10.1038/s41598-019-53517-3 (PMC6864243; doi:10.1038/s41598-019-53517-3)
Supplement: Supplementary file 1 — Supplementary information [file 41598_2019_53517_MOESM1_ESM.pdf]

Supplementary Information

Modes of the antibiotic activity of amphotericin B against *Candida albicans*

Ewa Grela[1,2], Agnieszka Zdybicka-Barabas[3], Bozena Pawlikowska-Pawlega[4], Malgorzata Cytrynska[3], Monika Wlodarczyk[1,4], Wojciech Grudzinski[1,] Rafal Luchowski[1] & Wieslaw I. Gruszecki[1,]**

1. Department of Biophysics, Institute of Physics, Maria Curie-Sklodowska University, Lublin, Poland
2. Department of Plant Physiology and Biophysics, Institute of Biological Sciences, Faculty of Biology and Biotechnology, Maria Curie-Sklodowska University, Lublin, Poland
3. Department of Immunobiology, Institute of Biological Sciences, Faculty of Biology and Biotechnology, Maria Curie-Sklodowska University, Lublin, Poland
4. Department of Functional Anatomy and Cytobiology, Institute of Biological Sciences, Faculty of Biology and Biotechnology, Maria Curie-Sklodowska University, Lublin, Poland.

* corresponding authors

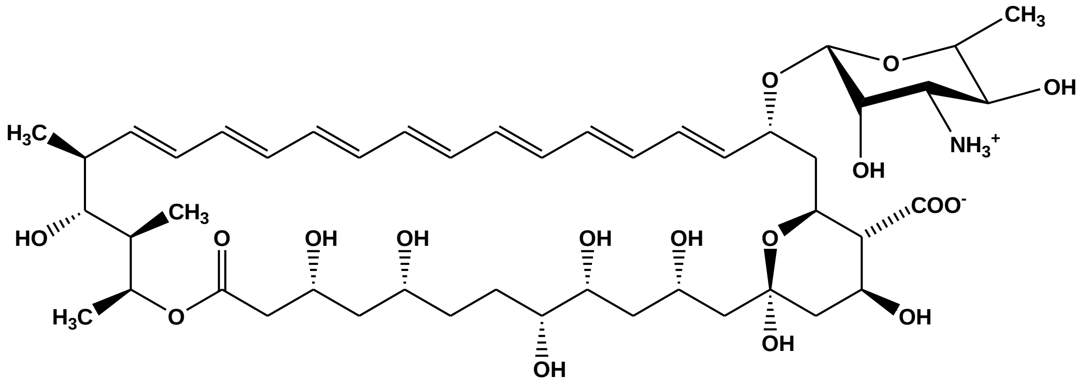

Figure S1. Chemical structure of amphotericin B molecule.

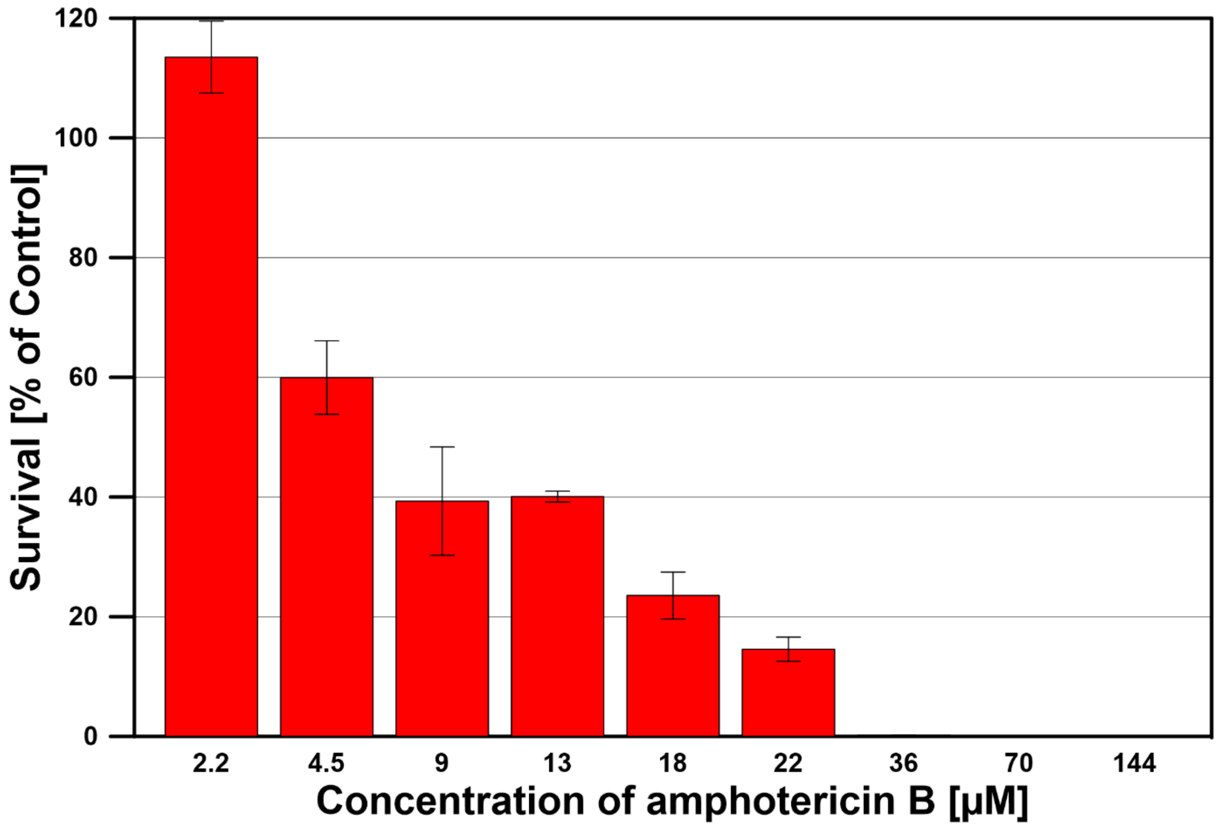

Figure S2. Results of survival assays of *Candida albicans* cells cultured under presence of amphotericin B. The results represent the mean of three independent experiments, each performed in triplicate ± S.D. Note the results at the 0 level for the concentrations 36 μM and higher.

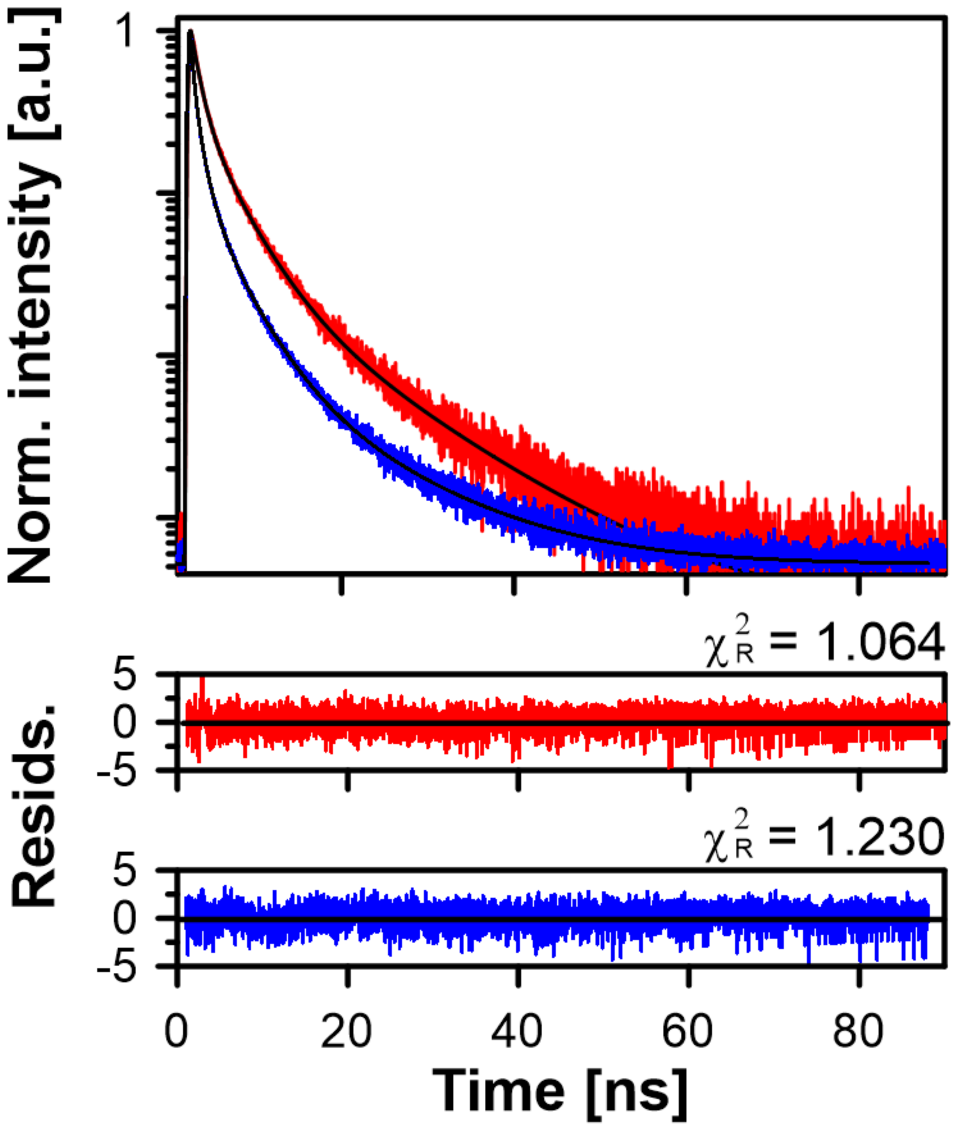

Figure S3. Fluorescence decay traces. Normalised fluorescence decays recorded for images presented in Figure 1 of the paper for time 0 (red) and 16 min after the exposition to AmB (blue). The background level of both the decays was 5 counts. The maximum number of counts was 8 400 for autofluorescence (0 min) and 42 000 for the picture with AmB addition (16 min). The goodness of fits is given above the residuals.

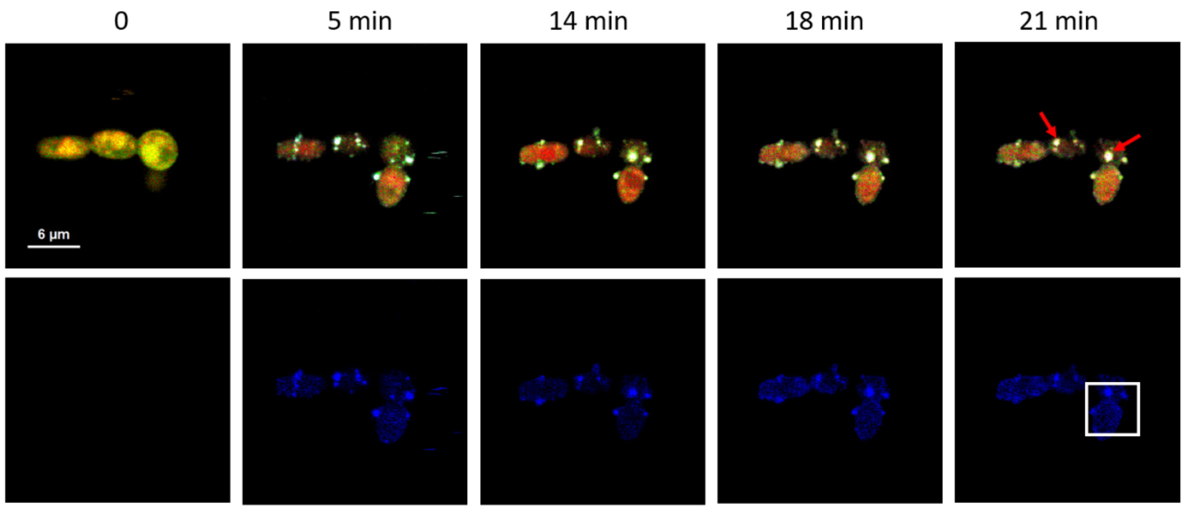

Figure S4. Fluorescence lifetime images of cells. Cells of *Candida albicans* were imaged with FLIM technique before (time 0) and after the injection of amphotericin B. Images were recorded after the time periods indicated at the top of each image. The colour codes of the images: blue - the fluorescence lifetime component 0.2 ns, green - the fluorescence lifetime component 1.0 ns, red – the fluorescence lifetime component 3.7 ns. The lower panel presents the same images at the top panel but with displayed exclusively the short-lifetime component representing the presence of AmB in the objects imaged. A final concentration of AmB in the sample 100 μM. Concentration of DMSO 0.3 %Note the appearance and time-evolution of the bright, bulk structures of AmB. Selected bulk structures that appeared after exposure of the cells to AmB are marked with red arrows. The fluorescence anisotropy image of the structures framed with the white square is presented in Figure S3.

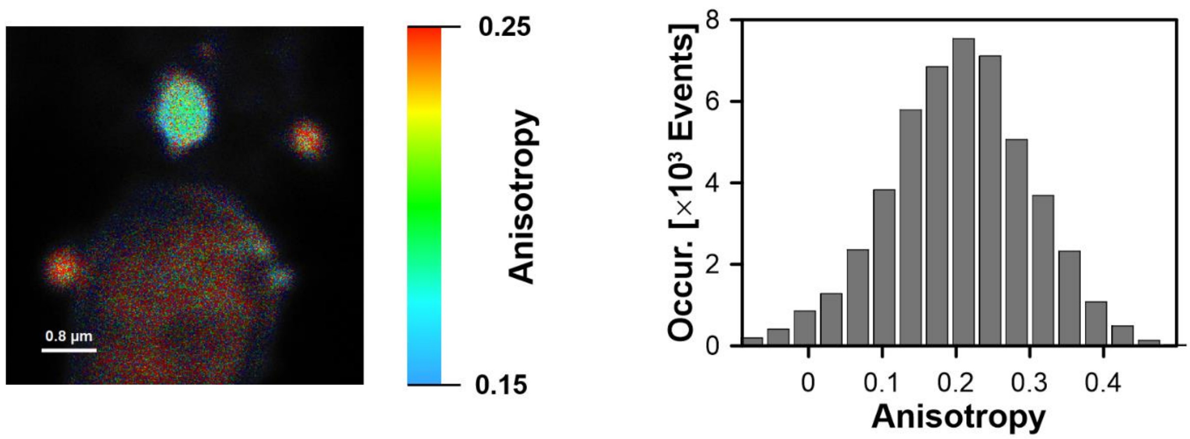

Figure S5. Fluorescence anisotropy image of cells and the extracellular structure.
A detailed image of the selected structures from the images of *Candida albicans* presented in Figure S3. The fluorescence anisotropy distribution analysis of the image is presented in the panel on the right-hand side.

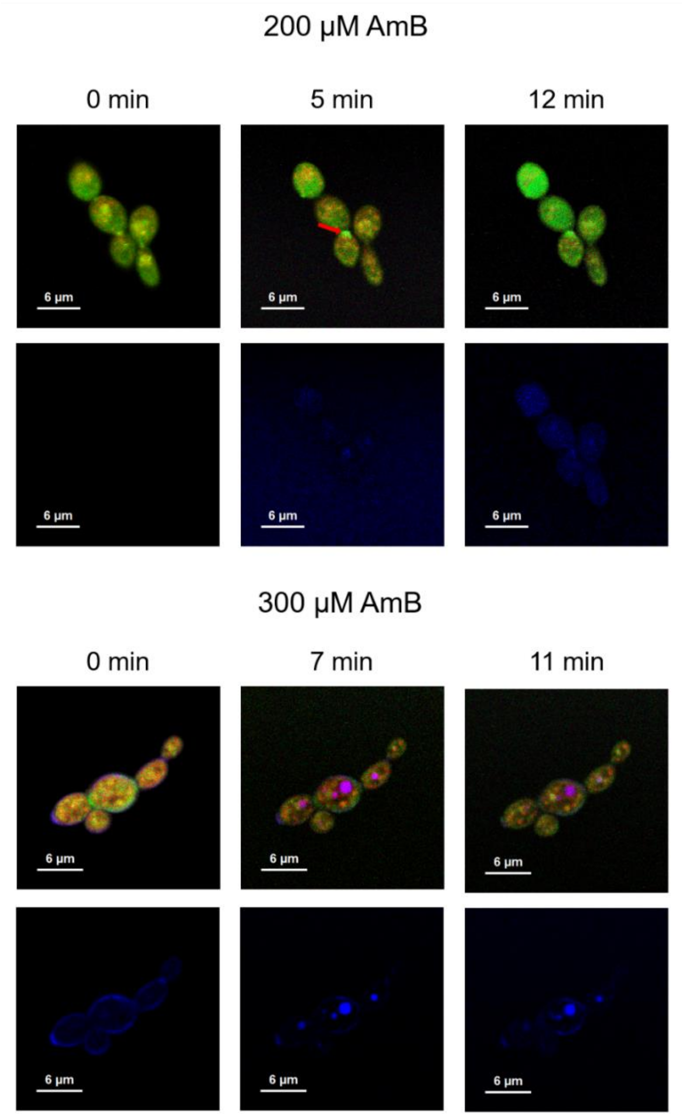

Figure S6. Fluorescence lifetime images of cells. Cells of *Candida albicans* were imaged with FLIM technique before (time 0) and after the injection of amphotericin B. Images were recorded after the time periods indicated at the top of each image. The colour codes of the images: blue - the fluorescence lifetime component 0.2 ns, green - the fluorescence lifetime component 1.0 ns, red – the fluorescence lifetime component 3.7 ns. The lower panels present the same images as the top panels but with displayed exclusively the short-lifetime component representing the presence of AmB in the objects imaged. The red arrow points the bulk structure that appears after the exposure of the cells to AmB. A final concentration of AmB in the samples was either 200 μM or 300 μM (indicated). The concentration of DMSO 0.3 %. Note the formation of extracellular bulk structures at a lower concentration of AmB (upper panels) and intracellular AmB-containing vesicles at a higher concentration of the drug.
